# Evidence Supporting Predation of 4-m Marine Reptile by Triassic Megapredator

**DOI:** 10.1016/j.isci.2020.101347

**Published:** 2020-08-20

**Authors:** Da-Yong Jiang, Ryosuke Motani, Andrea Tintori, Olivier Rieppel, Cheng Ji, Min Zhou, Xue Wang, Hao Lu, Zhi-Guang Li

**Affiliations:** 1 Laboratory of Orogenic Belt and Crustal Evolution, Ministry of Education; Department of Geology and Geological Museum, School of Earth and Space Sciences, Peking University, Yiheyuan Street. 5, Beijing 100871, People's Republic of China; 2 Department of Earth and Planetary Sciences, University of California, Davis, One Shields Avenue, Davis, CA 95616, USA; 3 Dipartimento di Scienze della Terra, Università degli Studi di Milano, Via Mangiagalli, 34-20133 Milano, Italy; 4 Integrative Research Center, The Field Museum, Chicago, IL 60605-2496, USA; 5 Nanjing Institute of Geology and Palaeontology, Chinese Academy of Sciences, Beijing East Road 39, Nanjing, Jiangsu 210008, People's Republic of China; 6 The Geoscience Museum, Hebei GEO University, No. 136 East Huai'an Road, Shijiazhuang, Hebei 050031, People's Republic of China

**Keywords:** Biological Sciences, Zoology, Paleobiology

## Abstract

Air-breathing marine predators have been essential components of the marine ecosystem since the Triassic. Many of them are considered the apex predators but without direct evidence—dietary inferences are usually based on circumstantial evidence, such as tooth shape. Here we report a fossil that likely represents the oldest evidence for predation on megafauna, i.e., animals equal to or larger than humans, by marine tetrapods—a thalattosaur (∼4 m in total length) in the stomach of a Middle Triassic ichthyosaur (∼5 m). The predator has grasping teeth yet swallowed the body trunk of the prey in one to several pieces. There were many more Mesozoic marine reptiles with similar grasping teeth, so megafaunal predation was likely more widespread than presently conceived. Megafaunal predation probably started nearly simultaneously in multiple lineages of marine reptiles in the Illyrian (about 242–243 million years ago).

## Highlights

• A new fossil of a 5-m ichthyosaur contains remains of a 4-m thalattosaur
• It likely represents the oldest record of megafaunal predation by a marine reptile
• More Mesozoic marine reptiles than previously conceived likely fed on megafauna
• Megafaunal predation simultaneously started in a few lineages of marine reptiles

## Graphical Abstract

**Figure:**
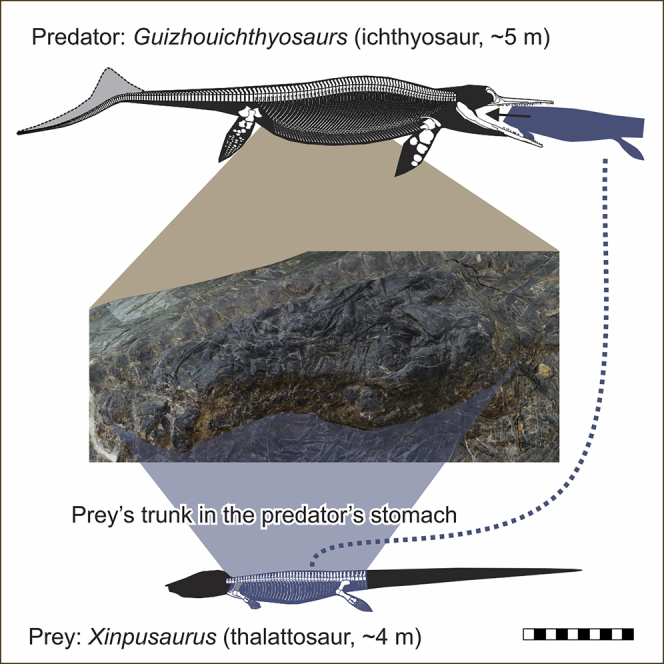

## Introduction

Land vertebrates started recolonizing the sea after the end-Permian mass extinction and diversified into many ecomorphs including apex predators (Kelley and Pyenson, 2015; Motani, 2009; Vermeij and Motani, 2018). Such predators are found among cetaceans and pinnipeds in the modern ecosystem but belonged to marine reptiles, such as plesiosaurs, mosasaurs, and ichthyosaurs, in the Mesozoic (Massare, 1987). However, direct evidence supporting the inference that some fossil marine reptiles preyed on marine megafauna is rare (Massare, 1987)—only five genera of Mesozoic marine reptiles have so far been reported with remains of other tetrapods in the stomach (Table 1), namely, three mosasaurs (*Tylosaurus,* Everhart, 2004; Konishi et al., 2014; Massare, 1987; *Hainosaurus,* Konishi et al., 2014; Russell, 1967; *Prognathodon,* Konishi et al., 2011) and two ichthyosaurs (*Temnodontosaurus,* Böttcher, 1989; McGowan, 1974; and *Platypterygius,* Kear et al., 2003). Even in these exceptional cases, only up to several bones of the prey, disarticulated and usually fragmentary, are preserved, and some of these prey tetrapods may not be large enough to be considered megafaunal species.

**Table 1:** Comparison of Appendicular Bone Lengths in the Prey in XNGM-WS-53-R4 with Those of the Holotype of *Xinpusaurus xingyiensis* (XNGM-WS-53-R3)

	Prey in XNGM-WS-53-R4	Holotype of *Xinpusaurus xingyiensis*	Ratio
Humerus	67+	60	NA
Radius	49	32	1.53
Femur	104	80	1.30
Fibula	55	41	1.34
Tibia	56	42.5	1.32
Ilium	92	75	1.23
Geometric mean	67.9	50.7	1.34^a^

a Ratio of geometric means, not geometric means of ratios.

In the absence of direct evidence, circumstantial evidence, such as body size and tooth shape, has been used to infer the diet of fossil marine reptiles (Massare, 1987). If a large species has large teeth with carinae (cutting edges), it is usually considered the apex predator of its ecosystem even in the absence of a direct record of its diet (Frobisch et al., 2013; Massare, 1987). However, it is also known that not all marine apex predators have teeth with carinae (Böttcher, 1989; Massare, 1987), e.g., the killer whale (*Orcinus orca*) and *Temnodontosaurus trigonodon*. Moreover, the largest marine vertebrate of a given ecosystem is often not the apex predator—the largest whales are filter feeders, and so were the largest Mesozoic fishes (Friedman et al., 2010). The largest Mesozoic marine reptile, belonging to ichthyosaurs, was edentulous (Nicholls and Manabe, 2004), suggesting it did not feed on large bony prey. Modern marine apex predators, such as the killer whale and great white shark (*Carcharodon carcharias*) are typically in the size range of 5–8 m (Mikhalev et al., 1981; Ralls and Mesnick, 2018) and 3–6 m (Boldrocchi et al., 2017; French et al., 2018), respectively, although the largest individuals of *O. orca* reach 9 m. There are larger whales, such as baleen whales (up to about 30 m), sperm whale (up to about 18 m), and beaked whales (up to about 12 m) (Jefferson et al., 1993), but they are not apex predators.

Ichthyosaurs are a group of Mesozoic marine reptiles that gave rise to a fish-shaped body profile. They belong to Ichthyosauromorpha (Chen et al., 2014; Jiang et al., 2016; Motani et al., 2015b), a clade that emerged in the late Early Triassic, approximately coinciding with the marine invertebrate diversification in the late Early Triassic (Fan et al., 2020; Fu et al., 2016; Motani et al., 2017). They radiated in the latest Early to Middle Triassic and became air-breathing top predators during the process of reconstruction of the Triassic marine ecosystem after the end-Permian extinction. The lineage gave rise to the oceanic clade Euichthyosauria, which later “dominated” the Mesozoic oceans in the Jurassic and Cretaceous. The evolution of their diet and feeding functions has previously been studied to some extent (McGowan and Motani, 2003; Motani et al., 2013; Scheyer et al., 2014). However, many questions remain as to which species of ichthyosaurs were truly apex predators.

*Guizhouichthyosaurus* is a genus of ichthyosaur that would not be considered an apex predator based on the traditional criteria. Its typical size range is about 4–6 m in total length, being smaller than the killer whale (*O. orca*), although some individuals reached about 7 m. Its teeth lacked carinae and were not very large for the body size. However, in 2010, a nearly complete skeleton of *Guizhouichthyosaurus* that directly contradicts this interpretation was excavated from the Ladinian (Middle Triassic) Zhuganpo Member of the Falang Formation in Xingyi, Guizhou, southwestern China. The specimen, spanning 4.8 m, was subsequently prepared in the laboratory of the Xingyi National Geopark Museum. In the abdominal region of this specimen, an obvious block of packed bones bulges above the bedding plane. After a detailed preparation, it can be discerned that the bones inside of this bulging block are not ichthyosaurian but originated from a thalattosaur, which was most likely hunted by the ichthyosaur. The prey remains are partially articulated and represent the trunk region without the skull or tail. The purpose of this article is to report the stomach contents and discuss their implications for the early evolution of megafaunal predation by marine tetrapods.

There is confusion in the literature regarding the term “macropredator.” There has been a recent tendency in the fossil marine vertebrate literature to call predators that feed on large vertebrates “macropredators” (Benson et al., 2013; Frobisch et al., 2013; Konishi et al., 2014). However, the same term has historically been used in the ecological literature to indicate macroscopic predators, many of which are not large. We therefore call the predators that feed on megafauna “megapredators”—the word megafauna traditionally refers to animals of adult human size (arbitrarily set at 44 kg) or larger (Stuart, 1991), although the threshold value may be as low as 10 kg in some studies (Wroe et al., 2004).

## Results

### Bromalite Features

**Figure 1:**
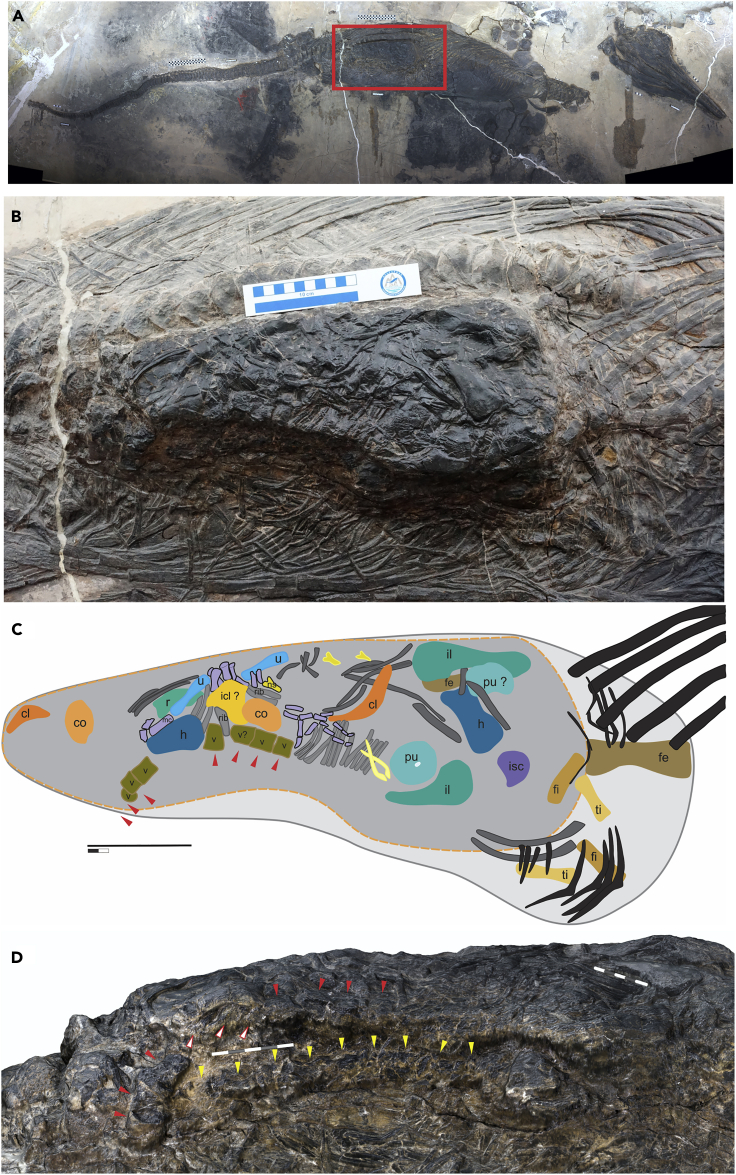
The Skeleton of *Guizhouichthyosaurus* (XNGM-WS-50-R4) and Its Stomach Contents (A) The skeleton. (B) Close-up of the stomach area, highlighted by red rectangle in (A). (C) Line drawing to show selected bone elements of prey in (B). (D) 3D rendering showing the ventral side view of the bromalite, revealing two strings of vertebrae. Red triangles point at the vertebrae that can been seen from the top surface, and white triangles with red outlines mark three vertebrae that are hidden by the humerus. Yellow triangles point to the vertebrae of the second string. Abbreviations: cl, clavicle, color in dark orange; co, coracoid, in light orange; fe, femur, in tan; fi, fibula, in goldenrod; h, humerus, in blue; icl, interclavicle, in yellow; il, ilium, in dark green; isc, ischium, in purple; mt, metacarpal, and all possible digit elements in light purple; ns, neural spine, in light yellow; pu, pubis, in light green; r, radius, in light green; ti, tibia, in khaki; u, ulna, in light blue; v, vertebral centrum, in dark gold. Bones in dark gray and gray, ribs of thalattosaur *Xinpusaurus xingyiensis*. Bones in black, ribs, and gastralia of ichthyosaur *Guizhouichthyosaurus* (XNGM-WS-50-R4). Scale bars, 25 cm in (A), 10 cm in (B and C), and 5 cm in (D).

The main specimen studied (XNGM-WS-50-R4) is of an ichthyosaur identified as *Guizhouichthyosaurus* (Methods), containing a dense concentration of many bones inside the ribs and gastralia in the abdominal region (Figure 1). These bones are considered a bromalite, a term that refers to all trace fossils representing food items that entered the oral cavity or gastrointestinal tract in life, whether or not they were expelled subsequently (Hunt and Lucas, 2012). These bones do not pertain to the skeleton of the ichthyosaur—they are morphologically different from those of the ichthyosaur, whereas the ichthyosaur postcranial skeleton is well-articulated, without any obvious lack of bones.

The entire bromalite is about 74 cm long anteroposteriorly and contains a central part that bulges beyond the bedding plane by about 15 cm. The bulge is approximately 47 cm long anteroposteriorly and 19 cm wide dorsoventrally. These anatomical orientations are based on the ichthyosaur that contains the bromalite, not the prey. Bones in the bromalite show little indication of etching by the stomach acid (see Discussion), whereas those exposed along the left wall have been physically damaged, together with the surrounding matrix, probably by a taphonomic cause.

The bromalite contains two vertebral strings with 10 and 9 vertebrae, respectively (Figures 1B–1D). Some neural arch elements are found disarticulated and scattered on the dorsal side of the bulge, and some ribs are preserved in an approximate series along the vertebral column. At least 13 types of appendicular bones are identified in the bromalite with confidence: the clavicle, coracoid, humerus, ulna, radius, metacarpal, manual phalanges, ilium, ischium, pubis, femur, tibia, and fibula (Figures 1B and 1C). In addition, a suspected interclavicle is also present. Some manual elements are preserved in partial articulation (Figures 1B and 1C). Most of the bones are found in pairs of the right and left elements with matching shapes and sizes.

### Prey Identity, Size, and Orientation

**Figure 2:**
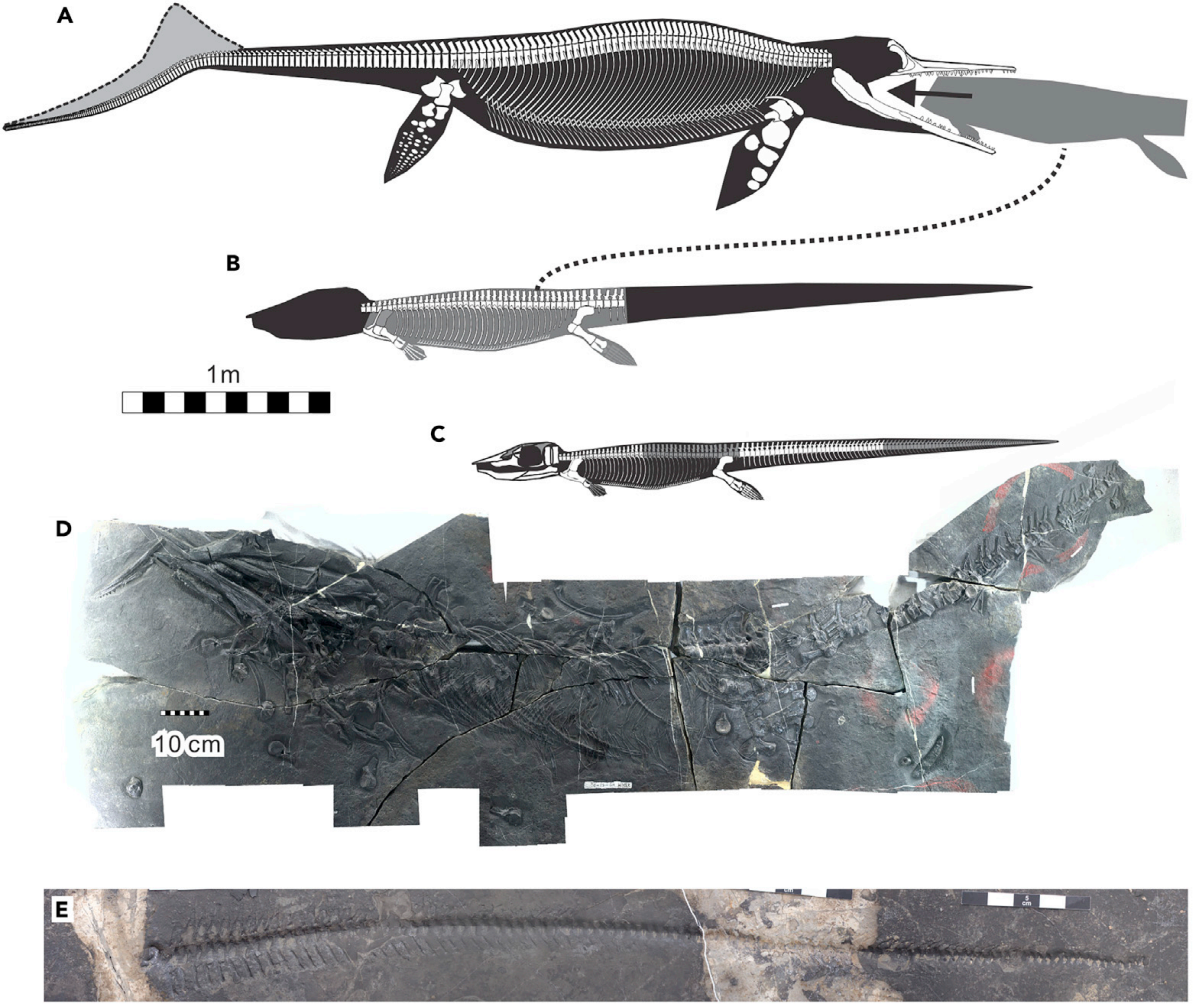
Body Configurations of the Predator and Prey in XNGM-WS-50-R4, when compared with XNGM-WS-53-R3 (A) Skeletal reconstruction of the predator, *Guizhouichthyosaurus*, in XNGM-WS-50-R4. (B) Approximate skeletal map of the prey, *Xinpusaurus xingyiensis*, in XNGM-WS-50-R4. (C) Skeletal reconstruction of the holotype of *X. xingyiensis* (XNGM-WS-53-R3). (D) Photographic view of *X. xingyiensis* (XNGM-WS-53-R3) with distortion removed by orthographic stitching. (E) A natural mold of an isolated tail of *Xinpusaurus* (XNGM-WS2011-50-R6) found 23 m away from XNGM-WS-50-R4, with the dorsal side facing below. Scale bars, 1 m for (A–C), 10 cm in (D), and 25 cm in (E).

**Figure 3:**
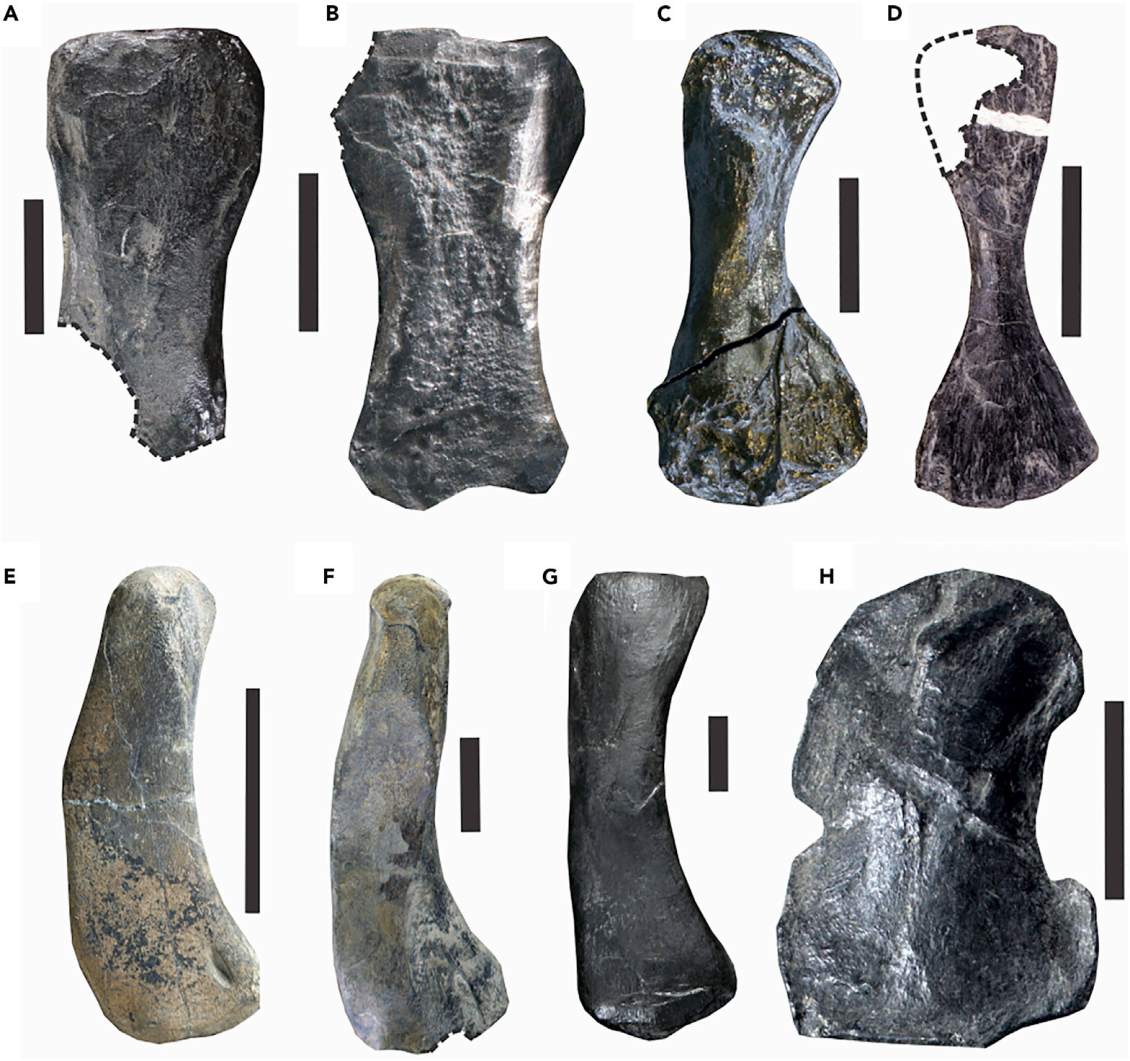
The Humerus of the Prey in XNGM-WS-50-R4, when compared with Those of Marine Reptiles from the Same Locality, as well as Coeval Localities in the Vicinity (A) Prey in XNGM-WS-50-R4. (B) *Xinpusaurus xingyiensis* (XNGM-WS-53-R3). (C) *Anshunsaurus wushaensis* (XNGM XY-2013-R2). (D) *Glyphoderma kangi* (XNGM XY-2016-R2). (E) *Lariosaurus xingyiensis* (XNGM-WS-30-R19). (F) *Nothosaurus youngi* (XNGM-WS-30-R24). (G) *Yunguisaurus liae* (XNGMXY-2013-R1). (H) *Qianichthyosaurus xingyiensis* (XNGM-WS-46-R1). Scale bars in black are 2 cm.

The prey is identified as the thalattosaur *Xinpusaurus xingyiensis* (Figure 2) based on close similarities of appendicular skeletal elements in both shape and size. The similarity is most characteristically seen in humeral morphology—it is a robust bone with a limited shaft constriction, and with an expanded proximal extremity (Figure 3A). The same morphology is found in the holotype of *X. xingyiensis* (Li et al., 2016) (Figure 3B) but not in any other marine reptiles of comparable sizes from the same locality or coeval localities nearby: the thalattosaur *Anshunsaurus wushaensis* has a humerus with a strongly constricted shaft and remarkable distal expansion (Figure 3C); eosauropterygians, such as *Lariosaurus* (Figure 3E) (Lin et al., 2017), *Nothosaurus* (Figure 3F) (Ji et al., 2014), and *Yunguisaurus* (Figure 3G) (Wang et al., 2019) have curved humeral shafts; the placodont *Glyphoderma* has a strongly constricted humerus (Figure 3D); and ichthyosaurs, such as *Qianichthyosaurus* (Figure 3H) (Yang et al., 2013) and *Guizhouichthyosaurus* (the present specimen), have humeri that are short and robust, with an anterior flange. Other bones also conform with the species identification. The femur is approximately rectangular and has a nearly straight distal end, as in *X. xingyiensis* (Li et al., 2016). The radius has an anterior flange, which is a character only seen in some thalattosauroids among thalattosaurs (Nicholls, 1999), including *X. xingyiensis* (Li et al., 2016). None of the bones in the bromalite shows features that contradict the species identification.

The appendicular bones of the prey are about 1.34 times longer than corresponding elements of the holotype of *X. xingyiensis* (XNGM-WS-53-R3) based on the geometric means of bone lengths (Table 1). The holotype has a preserved length of 2.1 m, excluding half of the tail that is missing (Figure 2D). Using the body proportion of the holotype of *Xinpusaurus suni*, a congeneric species that is slightly younger than *X. xingyiensis*, the total length including the missing half of the tail was estimated to be greater than 3 m (Li et al., 2016) (Figure 2C). When multiplying this value by 1.34, the prey thalattosaur is estimated to be about 4 m in total length (Figure 2B). Unfortunately, the sample size of authentic congeneric skeletons is too limited to allow incorporation of allometry to this estimation process.

There is no evidence to suggest that more than one individual prey is in the stomach of the predator: the bromalite does not contain excess bones beyond the number expected in a reptile, while the right and left bones have nearly identical sizes. The bones preserve the original skeletal association, with pectoral elements found posteriorly in the bromalite, and pelvic elements anteriorly, with only two pectoral bones displaced anteriorly. Thus, the posterior side of the bromalite is craniad for the prey. The orientation of the ribs also matches this prey orientation.

The skull, mandible, and tail of the prey are unlikely to be present in the bromalite, given that no isolated elements from these body regions are mixed in with what is preserved. Also, there is insufficient space in the bromalite to hide these parts—for example, the skull of the holotype of *X. xingyiensis*, which is smaller than the prey individual, would occupy most of the bromalite volume.

### Predator Morphology and Diet Suggested by Teeth

The body length of the predator skeleton along the vertebral column is about 4.80 m, after excluding the taphonomic gap in the neck. Although this is only 1.2 times that of the prey, body mass is expected to be roughly seven times greater in the predator because the body trunk diameter relative to body length is about 2.5 times greater—the prey is slender and elongated, whereas the predator is spindle shaped (Figure 2). It is an adult or at least young adult individual based on the degree of ossification of the humeral head (Johnson, 1977).

**Figure 4:**
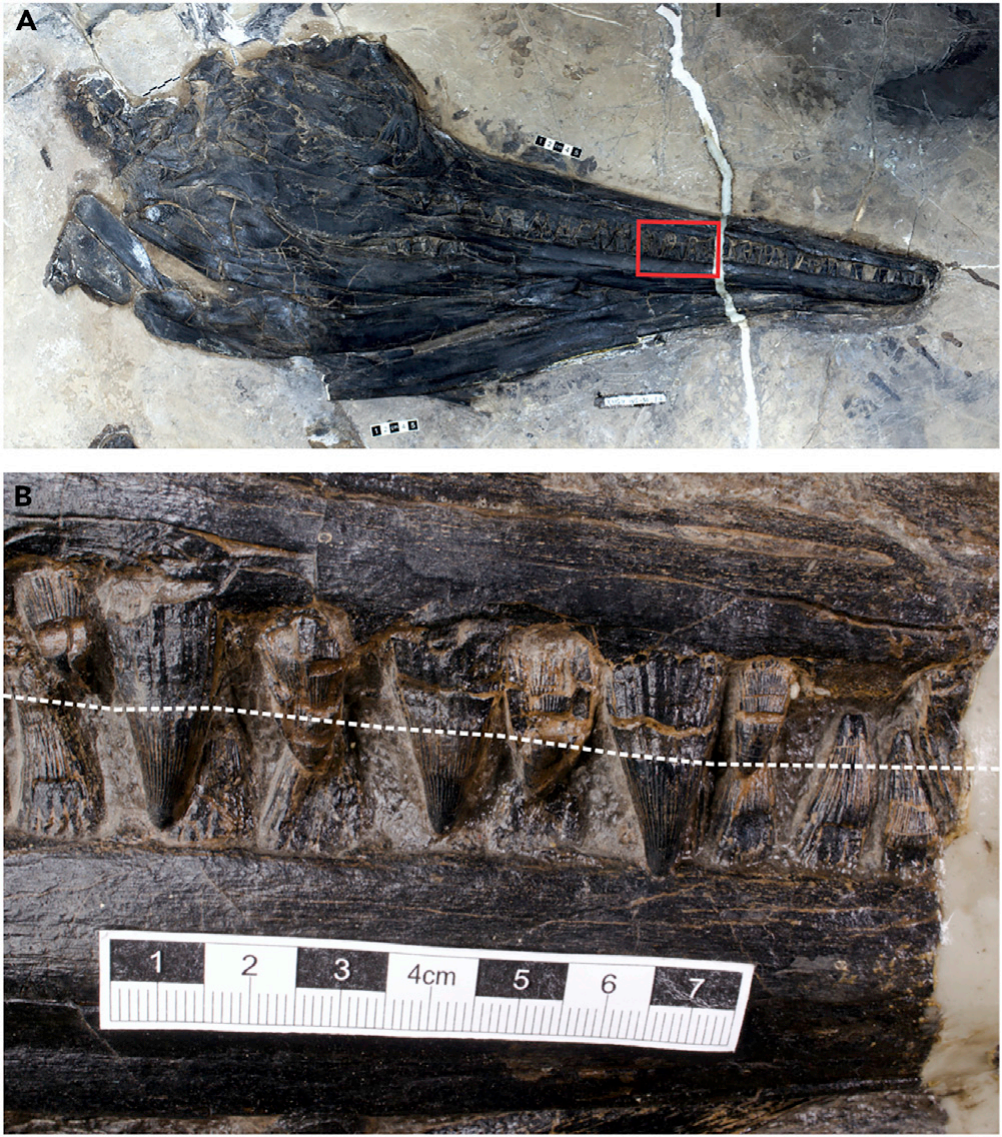
Skull and Dentition of the Predator in XNGM-WS-50-R4 (A) The skull. (B) Close-up of the dentition from the area with the red rectangle in (A) White broken line in (B) indicates an approximate position of the gum line for the upper jaw. Note that the tooth parts that are exposed beyond the gum line are small and not densely packed. Scale bars are 5 cm in (A) and 7 cm in (B).

The teeth are well preserved in the closed mouth (Figure 4A). When considering that the gum reached the boundary between the crown and root of fully erupted teeth, parts of the teeth that were exposed in life were not tightly packed (Figure 4B). All teeth are almost uniformly conical and bluntly pointed, without cutting edges. The largest teeth are found in the posterior-most premaxillary and anterior-most maxillary regions (Figure 4B), among which the largest is 28.0 mm long and 15.8 mm wide at the base (crown height 15.6 mm, base width 9.4 mm). The relative tooth size index based on the tooth crown height relative to the skull width (about 20.4 cm) is about 0.086, and the tooth shape index is 1.51 based on the tooth crown height relative to the width.

Massare proposed that three characteristics of the dentition were correlated with prey preference of marine tetrapods, i.e., relative size to the skull width, crown aspect ratio, and tooth wear (Massare, 1987). The tooth size index places the present ichthyosaur in either the Crunch or Smash guild (collectively called grasping teeth). The crown shape index points to the Crunch guild, whereas the apices of the teeth, being unpolished and unbroken, suggest the Smash guild. In such cases of conflicting outcomes, Massare suggested that the relative tooth size and apex shape were more important than the crown shape index (Massare, 1987). Thus, the ichthyosaur belongs to the Smash guild. The teeth of this guild are “used for grasping fairly soft prey such as belemnoids and soft cephalopods” (Massare, 1987).

### Isolated Tail

An isolated *Xinpusaurus* tail (XNGM-WS2011-50-R6) was found near the main specimen (XNGM-WS-50-R4). The generic identification is based on the morphology of mid-caudal centra—of the two thalattosaurs from coeval localities in the region, *A. wushaensis* has anteroposteriorly elongated mid-caudal centra, whereas these centra are slightly higher than long in *X. xingyiensis*, as in the present specimen. The tail is about 2 m long along the vertebral column, containing 96 to 97 vertebrae (Figure 2E). Given that *Xinpusaurus* has up to 100 caudal vertebrae (Liu, 2015), this caudal series should represent a nearly complete tail, probably missing the first few vertebrae. The centra are tightly articulated with each other, except the most anterior one that is preserved in an inclined posture.

## Discussion

The main specimen (XNGM-WS-50-R4) likely represents the oldest direct record of megafaunal predation by marine tetrapods and also sets the record for the largest prey size of Mesozoic marine reptiles at 4 m (Table 2), which is larger than the previous record of 2.5 m (Everhart, 2004; Konishi et al., 2014). This statement disregards an uncertain estimation given in an unpublished dissertation, where a partial series of gastralia and a few vertebrae of a brachaucheniid pliosaur, presumably belonging to *Kronosaurus* based on its geologic setting (i.e., Lower Cretaceous of the Great Australian Basin), was reported in association with seven elasmosaurid vertebrae. The total length of this elasmosaurid was estimated to be 3.3 m based on the body proportion of an elasmosaurid species from the same basin but could be bloated up to 5.0 m if a longer-necked species from North America was used as the model. The fragmentary nature of the specimen obscures if the presumed prey was in the stomach of the postulated predator. Although fish prey are not included in Table 2, fish found in marine reptile bromalites have so far been smaller than 2.5 m in total length (Konishi et al., 2014). The comparisons here are limited to lengths because it is difficult to estimate body masses accurately based on flattened fossils.

**Table 2:** Published Records of Tetrapod Remain in the Stomach of Mesozoic Marine Reptiles, Excluding an Abandoned Embryo that was Obviously Scavenged

Predator		Prey Tetrapod			Ref.
Taxon	Size	Taxon	Body Parts	Total Length	
*Tylosaurus proriger*	Total 8.8 m skull 1.2 m	*Dolichorhynchops* sp. (plesiosaur)	Partial humerus, 4 caudal vertebrae, 3 caudal ribs	∼2.5 m	Everhart (2004)
	Unknown	*Latoplatecarpus* sp. (mosasaur)	Unknown	>2 m	Konishi et al. (2014); Massare (1987)
	Unknown	*Hesperornis* (stem bird)	Unknown	Unknown	Konishi et al. (2014); Massare (1987)
*Hainosaurus* sp.	Unknown	Stem sea turtle	Unknown	Unknown	Konishi et al. (2014); Massare (1987); Russell (1967)
*Prognathodon overtoni*	total 5.9 m	*Nichollsemys baieri* (stem sea turtle)	Jugal, postorbital, quadrate, supraoccipital	0.6 m carapace length	Konishi et al. (2011)
Pliosauroid (probably *Kronosaurus*)	Total ∼10.5 m	Elasmosauroid	7 vertebrae	3.3(-5.0?) m	McHenry (2009)
*Temnodontosaurus trigonodon*	Total 8.7 m	*Stenopterygius* sp. (ichthyosaur)	Vertebrae	Unknown	Böttcher (1989)
*Temnodontosaurus eurycephalus*	Skull 1.02 m	Ichthyosaur	Basioccipital	Unknown	McGowan (1974)
*Platypterygius australis*	Skull ∼0.4 m	Hatchling protostegid (stem sea turtle)	Isolated bones	100–120 mm	Kear et al. (2003)
		*Nanantius eos* (stem bird)	Proximal tibiotarsus	Unknown	Kear et al. (2003)
*Guizhouichthyosaurus sp.*	Total 4.8 m	*Xinpusaurus xingyiensis*	Body trunk	∼4 m	This work

Two questions arise concerning how the individual of *Xinpusaurus* found its way to the stomach of *Guizhouichthyosaurus*: was it by predation or scavenging, and, if the latter, were the head and tail detached by the scavenger or through postmortem decay? The answer to the second question is simpler than that for the first. Forensic taphonomy in the marine context has shown that hands and feet are the first to be detached through postmortem decay of human remains at sea, followed by the head and then more proximal parts of the limbs, whereas the vertebral column is the last to disintegrate, being strongly reinforced by extensive connective tissues that take time to decay (Haglund, 1993; Mason et al., 1996). This tendency is expected to have been exaggerated in *Xinpusaurus*, which likely used its body axis for propulsion, whereas its small limbs were used as rudders without a role of body support; appendicular connective tissues must have been limited relative to those along the axial skeleton, allowing faster decay. Then the presence of at least one manus and some pedal elements in the absence of the head and tail therefore cannot be explained very well by the decay hypothesis.

Possibilities of predation versus scavenging merit careful consideration. We conclude that predation is more likely than scavenging for the following reasons. First, marine carrion usually results from partial predation rather than deaths due to other causes (Barrett-Lennard et al., 2011; Britton and Morton, 2004). If a predator other than *Guizhouichthyosaurus* killed the thalattosaur in question, then it would be strange for the nutritious trunk and limbs to be left intact by the predator. Second, ingestion likely took place at the sea surface where *Guizhouichthyosaurus* was able to breathe because swallowing of a large food item would have taken a long time, whether the food was ingested in one or a few pieces. This would limit the possibilities of scavenging because marine scavenging usually occurs at the seafloor (Britton and Morton, 2004; Whitehead and Reeves, 2005)—marine carrion usually do not stay afloat at the surface (Haglund, 1993; Mason et al., 1996). In addition, the specimen is from the subtropical region of the warm Middle Triassic period, so the decomposition would have been rapid, further narrowing the window of time when carcass would have been available at the sea surface. Third, marine carrions are rare (Britton and Morton, 2004), especially that of megafauna available within the diving depth of typical air-breathing predators like killer whales (Whitehead and Reeves, 2005) and *Guizhouichthyosaurus*.

Even in the unlikely case of the present bromalite representing scavenged prey, *X. xingyiensis* would still be on the list of prey actively hunted by *Guizhouichthyosaurus.* Obligate scavenging by large animals is rare in modern marine ecosystems (Beasley et al., 2012; Wilson and Wolkovich, 2011), instead, marine scavenging is almost always facultative (Britton and Morton, 2004; Hammerschlag et al., 2016). Modern megapredators, such as the great white shark (*Carcharodon carcharias*) (Tucker et al., 2019), tiger shark (*Galeocerado cuvier*) (Hammerschlag et al., 2016), and killer whale (*O. orca*) (Whitehead and Reeves, 2005), are known to scavenge when given opportunities, but they tend to scavenge the carrion of the species that they also hunt. The carrion that they scavenge is derived from predation by the same or another individual, unless it is human caused (Britton and Morton, 2004).

The isolated tail specimen (XNGM-WS2011-50-R6) also supports the predation hypothesis. The specimen witnesses, whether it belonged to the prey individual in the bromalite or not, that there was a mechanism to detach the tail of a large thalattosaur while it was intact—decay was probably not involved because the distal part of the tail is still articulated, whereas decay would have detached that region first because there is less connective tissue there. The specimen instead shows that the most proximal vertebra, which would be the last to decay, is halfway detached (Figure 2E). External forces would be necessary to cause such detachment, and it is difficult to find a source outside of a predator. Thus, there was at least a predator that could hunt *Xinpusaurus*, whereas *Guizhouichthyosaurus* was the only species larger than the prey in this and coeval localities in the region.

Circumstantial evidence suggests that the isolated tail belongs to the prey individual in the bromalite. The tail was only 23 m away from the ichthyosaur specimen on the same rock surface and has a size and completeness that are expected for the lost tail of the prey in the bromalite. Also, as stated above, its preservation suggests that the tail was detached from the body while it was intact. If it is from the prey individual, the predator likely died soon after ingesting the prey, and that may explain the lack of etching of the bone in the bromalite by the stomach acid, as well as the strange detachment of the neck of the predator. Unfortunately, it is impossible to test this hypothesis directly so a clear conclusion cannot be drawn on this issue.

The mechanism that the ichthyosaur would have used to remove the head and tail of the thalattosaur before ingestion is not directly recorded in the fossil. However, it most likely employed the “grip and tear” strategy, which is the only method used by extant aquatic megapredators to separate the prey into pieces (Hocking et al., 2017). In this method, the prey is torn apart by a combination of the physical inertia of the prey's body and torsion applied by the predator's jaws through jerking and twisting while holding a part of the prey in the jaws. The method is used by the killer whale and leopard seal among marine mammals (Hocking et al., 2017), and crocodiles among reptiles (Ng and Mendyk, 2012; Pooley and Gans, 1976). Bottlenose dolphins are also known to use similar behaviors to tear prey (Sprogis et al., 2017), although they are not megapredators. None of these predators use cutting teeth when tearing the prey, i.e., the leopard seal uses the canine and peg-like incisors but not the carnassial, whereas others lack cutting teeth. The teeth of *Guizhouichthyosaurus* are robust and not especially large for the skull width, as in crocodiles that employ the “grip and tear” strategy. The ichthyosaur may have lacked the strong pterygoideus muscle typical of crocodiles, but its myology is too poorly understood to enable a fruitful discussion of muscular force that held down the prey. Also, the bite force of *Guizhouichthyosaurus* is expected to be large by virtue of size alone—simple isometric geometry suggests that the bite force would quadruple when the body length is doubled, and the observed increase of bite force with size in *Alligator* is even greater (Erickson et al., 2003). The fact that the prey vertebral column is broken into strings suggests that *Guizhouichthyosaurus* indeed had bite force needed for that task. About 40 vertebrae are expected be present in the bromalite, judging from the typical vertebral counts of *Xinpusaurus* (Liu, 2015). Given that the vertebrae in the bromalite are found in strings of about 10 each, it is likely that the prey was swallowed in one to about four pieces. It is possible that the prey's trunk was in one piece with the vertebral column broken into three to four strings inside because the distance between the overlapping parts of the two vertebral strings in Figure 1D is as short as 1.5 cm—it would be difficult to pack them that closely if the strings had been swallowed as parts of difference pieces, with each string surrounded by connective tissues that would prevent them from lying closely together. The observed arrangements may instead be reached by swallowing a single mass of connective tissues with the vertebral column broken into strings inside, the vertebral strings may shift and overlap partly as the mass was pushed into the digestive system. This would be especially the case if the time between ingestion and predator's death is short, not leaving much time for digestion.

Ingestion of the prey may have been aided by a combination of inertial feeding and placement of the prey above the water to utilize gravity with least counteraction from buoyancy (Gans, 1969; Hocking et al., 2017). The use of gravity to aid ingestion is known among marine mammals (Hocking et al., 2017), as well as terrestrial ingestion of large prey by *Varanus* (Editorial, 2018). The throat and neck muscles of ichthyosaurs are poorly understood, so it is difficult to judge how these muscles could aid in ingestion of large prey pieces. Ichthyosaurs lack cranial and mandibular kinesis that allows some modern reptiles to swallow large prey, but it is evident from the bromalite contents that the ichthyosaur predator could let the prey pass through the intermandibular space even without such kinesis. The shoulder girdle of *Guizhouichthyosaurus* is small relative to the body, unlike in basal ichthyopterygians or mixosaurs, leaving the front side of the rib cage widely open without bony obstruction. This may have helped allow passage of large prey pieces into the digestive system.

As described earlier, three characteristics of the dentition suggest that the *Guizhouichthyosaurus* specimen belonged to the Smash feeding guild, whose members prey on soft cephalopods (Massare, 1987). In contrast, the bromalite reveals that its diet included large marine reptiles. The widely used tooth-based diet estimation scheme (Massare, 1987) captures the majority trend well, but taxa that are not fully accounted for have been noted before (Konishi et al., 2011; Motani, 1997, 1996; Pierce et al., 2009). Two of these counterexamples concerned taxa with grasping teeth, and the present case adds to the list. Therefore, the tooth-based scheme may underestimate the extent of megafaunal predation by marine tetrapods as suspected before.

Although grasping by teeth is typically thought to be effective when feeding on cephalopods and fish, it also constitutes an effective mechanism when feeding on air-breathing prey in water. The predator can grasp the prey in the mouth and hold it underwater until it weakens through the lack of oxygen, as seen in the hunting strategy of some crocodiles (Ng and Mendyk, 2012). The prey runs out of oxygen before the predator because it struggles to escape while the predator stays still. Moreover, scaling effects help the predator because heavier tetrapods tend to be able to hold their breath longer (Stephens et al., 2008). Once the prey weakens, the predator can start the process of prey size reduction and ingestion without much resistance from the prey, taking advantage of the gravity above water and inertia in water (Gans, 1969; Hocking et al., 2017). Therefore, there may have been more megapredators with grasping teeth than currently recognized. Candidates include two ichthyosaur genera from the Illyrian (Anisian, Middle Triassic) with grasping teeth and slightly larger body sizes than *Guizhouichthyosaurus*: *Besanosaurus* (Dal Sasso and Pinna, 1996), a monotypic genus that belongs to Shastasauridae (as does *Guizhouichthyosaurus,* Ji et al., 2016), and *Cymbospondylus* (Merriam, 1908).

*Besanosaurus* has a small skull and teeth for the body with a slender snout and has hitherto been considered a cephalopod feeder (Dal Sasso and Pinna, 1996). However, its skull is large enough to hold a coeval ichthyosaur, such as *Mixosaurus cornalianus* Type B (Brinkmann, 1998a, 1998b) and *Mixosaurus. kuhnschnyderi* (Brinkmann, 1998a, 1998b). The type specimen contains isolated ichthyosaur vertebrae that were originally interpreted as embryonic remains (Dal Sasso and Pinna, 1996). Although this interpretation may be correct, it is possible that the vertebrae represent parts of a prey *Mixosaurus* because the vertebral centra are too well developed for an embryo of the developmental stage indicated by their size: the centra are fully developed without a notochordal pit in the middle, whereas they are between 55% and 75% of the expected terminal size (i.e., the longest vertebrae are about 13% of the longest adult vertebrae, when newborn ichthyosaurs are about 18%–23% of adult lengths, Motani et al., 2014; O'Keefe and Chiappe, 2011).

Currently, the cymbospondylid *Thalattoarchon* from the Illyrian (about 243–242 million years ago), known only from a partial skull, is considered the first marine tetrapod megapredator based on tooth morphology and an estimated body size (Frobisch et al., 2013). However, it is now likely that megafaunal predation by marine tetrapods started almost simultaneously at least among three genera within Cymbospondylidae and Shastasauridae during the Illyrian. Starting from small predators with a variety of diet and feeding styles (Cheng et al., 2019; Motani et al., 2015a, 2015b; Rieppel, 1998) in the Olenekian (Motani et al., 2017), Mesozoic marine tetrapods gave rise to multiple megapredators in about 7 million years, as rebuilding of biodiversity after the end-Permian mass extinction neared its completion (Chen and Benton, 2012).

### Limitations of the Study

The data for the present study are limited to what have been preserved in fossils. Inferences were made by interpreting such data in the context of modern anatomy, ecology, and taphonomy. As such, whereas the conclusions given represent most likely interpretations, they are not definitive.

### Resource Availability

#### Lead Contact

Further information and requests for resources should be directed to and will be fulfilled by the Lead Contact, Ryosuke Motani (rmotani@ucdavis.edu).

#### Materials Availability

The specimen studied is accessioned at the Xingyi National Geopark Museum, Xingyi City, Guizhou Province, China, with an accession number XNGM-WS-53-R4. It is presently on display for public viewing.

#### Data and Code Availability

All data used in the study are included in this publication. The present research did not use any new codes.

## Methods

All methods can be found in the accompanying Transparent Methods supplemental file.

## Supplemental Information

Document S1. Transparent Methods
