# Supplementary material for: Evidence Supporting Predation of 4-m Marine Reptile by Triassic Megapredator
Source: iScience. 2020 Aug 20;23(9):101347. doi: 10.1016/j.isci.2020.101347 (PMC7520894; doi:10.1016/j.isci.2020.101347)
Supplement: Document S1. Transparent Methods [file mmc1.pdf]

iScience, Volume *23*

Supplemental Information

Evidence Supporting Predation of 4-m

Marine Reptile by Triassic Megapredator

Da-Yong Jiang, Ryosuke Motani, Andrea Tintori, Olivier Rieppel, Cheng Ji, Min Zhou, Xue Wang, Hao Lu, and Zhi-Guang Li

TRANSPARENT METHODS

Method Details

Photogrammetry and Figures

Rendered image in Fig. 1D is based on a 3D surface model made with the photogrammetric software ReCap Photo 2020 Academic License from Autodesk, based on 76 photographs of the abdominal region of the main specimen. Scale bars were added in Meshlab 2020.4 (Cignoni et al., 2008). Figures were edited with CorelDraw 2018 Academic Edition.

Quantification and Statistical Analysis

Measurements below 17 cm were made with digital calipers that displays lengths down to 0.01 mm. Larger measurements were made with a tape measure with a millimeter scale. All calculations were made in R version 3.51 (R-Core-Team and R Core Team, 2020), in combination with Tinn-R version 5.01.02.00.

Additional Resources

The main fossil specimen for this study, XNGM-WS-50-R4 (Xingyi National Geopark Museum at Wusha, Xingyi, Guizhou Province, China), is an ichthyopterygian skeleton containing stomach contents (Fig. 1). It most likely represents a new species of *Guizhouichthyosaurus*, but its description is beyond the scope of this study. We instead refer to it by the genus name only. Its assignment to the genus is based on apomorphies, such as the pedal second digit with reduced proximal elements and well-developed distal phalanges, and a groove extending anteriorly from the external naris. The specimen, exposed in lateral to lateroventral view from left, is almost complete with a well-preserved skull, trunk, tail and flippers; only a few cervical vertebrae and some digital elements were missing (Fig. 1A). The stomach contents of

the specimen were compared with the holotype of the thalattosaur *Xinpusaurus xingyiensis* (XNGM-WS-53-R3), a well-articulated skeleton lacking the distal half of the tail (Fig. 2C, D) (*38*). We also examined an isolated tail of *Xinpusaurus* (XNGM-WS2011-50-R6; Fig. 2E).

All three specimens were excavated in 2010 in a quarry in Nimaigu, Wusha Town, Xingyi City, Guizhou Province, and belong to the Upper Assemblage of the Xingyi Fauna within the Ladinian (Middle Triassic) Zhuganpo Member of the Falang Formation. The outcrop has since become a field display of XNGM. The ichthyopterygian specimen XNGM-WS-50-R4 was found on the top surface of fossil bed number 50, which is a 55 cm thick laminated limestone layer, while the thalattosaur specimen XNGM-WS-53-R3 is from a 13-cm-thick limestone layer above, with bed number 53. These two beds are separated by only about 10 cm of limestone, therefore it can be inferred that these two species lived in the same geographical region without a significant temporal separation. Both fossils were mechanically prepared with pneumatic tools, and are deposited in XNGM. The tail specimen XNGM-WS2011-50-R6 is a natural mold exposed on the top surface of bed 50 at the field display of XNGM.
